# Effects of Diet on Brain Plasticity in Animal and Human Studies: Mind the Gap

**DOI:** 10.1155/2014/563160

**Published:** 2014-05-12

**Authors:** Tytus Murphy, Gisele Pereira Dias, Sandrine Thuret

**Affiliations:** Institute of Psychiatry, King's College London, The James Black Centre, 125 Coldharbour Lane, London SE5 9NU, UK

## Abstract

Dietary interventions have emerged as effective environmental inducers of brain plasticity. Among these dietary interventions, we here highlight the impact of caloric restriction (CR: a consistent reduction of total daily food intake), intermittent fasting (IF, every-other-day feeding), and diet supplementation with polyphenols and polyunsaturated fatty acids (PUFAs) on markers of brain plasticity in animal studies. Moreover, we also discuss epidemiological and intervention studies reporting the effects of CR, IF and dietary polyphenols and PUFAs on learning, memory, and mood. In particular, we evaluate the gap in mechanistic understanding between recent findings from animal studies and those human studies reporting that these dietary factors can benefit cognition, mood, and anxiety, aging, and Alzheimer's disease—with focus on the enhancement of structural and functional plasticity markers in the hippocampus, such as increased expression of neurotrophic factors, synaptic function and adult neurogenesis. Lastly, we discuss some of the obstacles to harnessing the promising effects of diet on brain plasticity in animal studies into effective recommendations and interventions to promote healthy brain function in humans. Together, these data reinforce the important translational concept that diet, a modifiable lifestyle factor, holds the ability to modulate brain health and function.

## 1. Introduction

One of the most remarkable capabilities of the brain is its ability to change in response to different stimuli. Among the highly sensitive, environment-responsive structures of the brain is the hippocampus, a region extensively known to regulate learning, memory, and mood [1–7]. Indeed, the expression of long-lasting activity-dependent synaptic modifications in response to stimuli of high frequency is an established phenomenon of the hippocampal neural circuitry [8]. This process of long-term potentiation (LTP) is considered as a prominent mechanism underlying learning and memory formation in the mammalian brain [9, 10]. Another hallmark of the hippocampus, in particular in its subregion which is called the dentate gyrus (DG), is the well-established capability of continual generation of new functional neurons throughout postnatal life. This process of adult hippocampal neurogenesis (AHN) is characterized by the presence of neural stem cells (NSC) with the ability to self-renew and differentiate into mature neurons [11] as well as by the microenvironment of the neurogenic niche, which engages the signalling pathways governing both proliferative activity and neuronal differentiation [12]. Functionally, AHN has been demonstrated to be essential for cognitive and emotional regulation, with its ablation or partial disruption, leading to severe impairment of learning abilities as well as increased depressive- and anxiety-related behaviors [13–17].

AHN is a highly regulated mechanism, meaning that the generation, migration, and integration of newly born neurons into preexisting circuits depend on complex signaling within the neurogenic niche [12]. NSC in the DG exist in close contact with local blood vessels [18]; this proximity is believed to aid the delivery of biochemical stimuli from the systemic milieu to the DG [19]. In turn, this can directly affect AHN for better, through the delivery of food-derived components to be used as precursors of neurotransmitters, or worse, by the exposure of the neurogenic niche to age-related inflammatory markers that inhibit neurogenesis [19].

A number of environmental factors have been shown to alter markers of brain plasticity, inducing changes not only in AHN [20, 21], but also of synaptogenesis [22], dendritic arborization [23], and spinogenesis [24], which in turn provide the biological substrate for adaptation to different environmental conditions [25, 26]. For instance, human stress reflects the increasing environmental challenges present in our society and actively modulates hippocampal structure and function, among other brain regions [27]. Indeed, animal studies have revealed that chronic stress reduces the dendritic tree of hippocampal neurons [28]. In contrast, voluntary exercise has been extensively shown to promote the local synthesis of growth factors, including brain-derived neurotrophic factor (BDNF) [21, 29] and to enhance AHN/angiogenesis [30].

Diet—encompassing total intake, frequency, and content—is also an important environmental factor that impacts brain plasticity, including AHN [31, 32]. Although there is much to be unravelled with regard to the specific molecular mechanisms through which dietary factors impact brain plasticity, a great body of literature supports the notion that diet modulates brain structure and function, exerting its influence throughout the lifespan of an organism. In this review, we primarily focus on studies from 2010 onwards that have investigated the effects of diet on markers of postnatal/adult brain plasticity, including AHN, trophic factors, and synaptic function. In particular, we review the effects of calorie restriction (CR), intermittent fasting (IF), polyphenols, and poly-unsaturated fatty acids (PUFAs) on the cellular and molecular mechanisms that underlie brain plasticity in context of cognition, mood/anxiety, aging, and Alzheimer's disease (AD) in both animal and human studies.

Although full coverage of all relevant studies is not possible given the broad scope of this review, the present work aims to evaluate the translational gap between the results from animal studies exploring the impact of diet upon markers of brain plasticity versus data from epidemiological and clinical intervention studies that suggest that diet influences both cognitive and emotional regulations across different disease contexts. Finally, we discuss some of the obstacles of harnessing the promising effects of diet on brain plasticity from animal studies into effective recommendations and interventions to promote healthy brain function in humans.

## 2. Calorie Restriction: Effects of Reducing What You Eat

### 2.1. Background and Physiological/Molecular Mechanisms of CR

The effects of CR—limiting calorie intake compared to baseline unrestricted or* ab libitum* (AL) consumption, with maintained levels of vitamin, mineral, or other essential biomolecules [33]—have attracted significant scientific attention since the pioneering work of McCay and colleagues showing that this intervention markedly extends the lifespan of rats [34]. In addition, CR was subsequently seen to entail significant benefits for “healthspan,” a term which describes the years of our life lived free of pathology and disease [35]. In this regard, CR has been shown to improve insulin sensitivity and autonomic function, as well as delay the onset of age-related process in many organisms ranging from yeast, worms, and flies [33] to higher mammals [36, 37]. Moreover, in human subjects, CR results in a multitude of health benefits [38], including reductions in abdominal fat mass, increased insulin sensitivity, and reduced levels of proinflammatory cytokines, reactive oxygen species, and atherosclerotic lipids in the blood [39, 40].

Theoretically, CR can be regarded as an example of hormesis, whereby too much of something evokes a detrimental response, whereas a smaller exposure but still above the normal range induces a mild stress that is in fact beneficial [38]. The signaling pathways involved culminate in the production of adaptive stress-response molecules that enhance the ability of the brain to resist more severe stress in the event of larger insults by promoting cell repair and survival, including trophic factors, antioxidant enzymes, DNA-repair enzymes,and proteins involved in mitochondrial biogenesis [38, 41]. It is likely that the beneficial effects of CR are a result of synergistic and/or additive effects of these multiple mechanisms.

It must be emphasised from the outset that CR is highly inappropriate for some groups of individuals, despite the putative benefits for metabolic health and brain plasticity discussed herein. In particular, CR is not a viable intervention for young children or pregnant women, where consistent adherence to a balanced diet is of paramount importance for physical development.

### 2.2. Impact of CR on Brain Plasticity

CR appears to both improve the resilience of synapses to metabolic and oxidative damage and modulate the total number, structure, and functional status of synapses [38]. In addition, maintenance on a CR regimen resulted in the differential expression of a multitude of genes, 25% of which were implicated in synaptic plasticity [42].

Interestingly, CR is associated with greater electrical and synaptic activity throughout neuronal circuits when compared to satiated and resting states [43]. In addition, CR stabilizes the levels of glutamate receptors and synaptic proteins required for excitatory transmission and thought to underlie hippocampal-dependent learning and memory [43, 44]. The generation of neurotrophic factors is another important adaptive and neuroprotective response to CR [45].

Table 1 outlines recent animal studies investigating the molecular players underlying the effects of CR on brain plasticity. For example, a role for the cAMP responsive-element binding-1 (CREB-1) has recently been suggested. Mice lacking the expression of this transcription factor in the forebrain were unable to exhibit the expected beneficial effects of CR on neuronal plasticity (enhanced LTP), memory (improved object recognition), and social behavior (reduced aggressiveness) when submitted to a 70% CR for a period of 5 weeks [46].

Long-term CR has also been shown to elicit working memory improvement in mice [47, 48]. In addition, significant increases in the expression of the NMDA receptor subunits NR2A and NR2B, essential for LTP and synaptic plasticity, were found in the hippocampus of 60% CR obese rats in comparison with age-matched AL-fed obese animals [49].

Whilst BDNF is a well-established molecule implicated in brain plasticity, no consensus exists regarding the upregulation of BDNF by CR. Whilst some argue in its favor [50, 51], others could not verify such an effect [52], a phenomenon that could be due to differences in the models used (aging, stroke, obesity, and others) and/or the duration and intensity of CR. In addition, when exercise training in conjunction with CR was applied to hypertensive rats, a synergistic effect between the two environmental interventions led to prevention of cognitive decline and upregulation of BDNF [51]. This suggests that the combination of CR with other positive inducers of brain plasticity, in this case exercise training, may provide more effective strategies to prevent cognitive decline [51].

### 2.3. Impact of CR on Mood/Anxiety

As discussed below in Sections 2.4.2 and 2.5.2, the majority of studies on the effects of CR in humans focused on the elderly population. For this reason, the findings reported in this section refer to animal studies only.

*Depression/Anxiety and Brain Plasticity*. Although very often reported as comorbid, depression and anxiety disorders are classified as distinct categories of neuropsychiatric illness. Depression is a mood disorder, along with bipolar disorder. It is a chronic illness characterized by persistent feelings of sadness and loss of interest in previously enjoyed activities [53]. In animal models, depressive-like behavior has been associated with decreased levels of AHN [14, 54]. In addition, in both animal and humans, depressive phenotypes have been consistently associated with altered levels of BDNF, suggesting that neural plasticity is significantly affected in the depressed brain. Before we discuss these data, we first describe depression and anxiety in context of different markers of brain plasticity.

Anxiety disorders are characterized by intense fear and autonomic responses to perceived or real threats. Neuroimaging studies have revealed that an extensive network of brain circuits is implicated in the generation of overexaggerated responses to potentially dangerous stimuli [55, 56]. At the cellular and molecular levels, decreased levels of AHN [17] and altered levels of BDNF [56] have also been associated with increased anxiety and may possibly account for their high comorbid prevalence with depressive disorders. Encouragingly, different dietary interventions hold the promising ability to reverse these changes in markers of brain plasticity [57].

#### 2.3.1. Animal Studies

Table 2 outlines recent animal studies on the effects of CR over mood/anxiety. Interesting findings in rodents were recently presented by Riddle et al. [58]. In their study, female adolescent mice and adults of both sexes that underwent 60% CR for only 7 days exhibited enhanced fear extinction learning and retention, a process normally impaired in patients with anxiety disorders [59] and which requires active neural plasticity [60]. Further to this, the effects observed in the CR mice could not be observed in age-matched serotonin transporter (SERT) knockout mice, suggesting that CR facilitates improved fear extinction through mechanisms that are SERT dependent [58]. In contrast, another study of lifelong 60% CR led to anxiogenic, rather than anxiolytic, effects [47]. Similarly, 50% CR when applied to young rats (starting at postnatal day 28 for 5 weeks) led to increased depression- and anxiety-like behaviors, which were accompanied by decreased expression of serotonin reuptake transporter [61]. This divergence among animal studies investigating the impact of CR on mood and anxiety could be due to differences not only in the duration of the dietary intervention, but also in the age and strain of animals. These incongruences highlight the need for further studies investigating the ideal contexts where CR could lead to promental health effects.

Lutter and colleagues report that the hypothalamic orexigenic hormone ghrelin has a major defensive role against depressive-like symptoms associated with chronic stress [62]. Consistently, 10 days of CR enhanced the activation of orexin cells after social defeat and reversed the behavioral deficits seen in wild-type mice submitted to the social defeat model of chronic stress [63]. This reversal was not observed in orexin knockout mice, revealing an additional candidate mechanism through which CR exerts its anxiolytic and antidepressant effects [63]. In contrast, exposure to moderate CR for 3 weeks led to an increased stress response [64]. Moreover, transition from CR to high-fat diet led to increased binge eating, an undesirable and stress-related outcome likely mediated by a CR-induced reprogramming of orexinergic pathways [64]. Together these results not only indicate that orexinergic pathways are another mechanism engaged by CR, but also point for the need of further studies addressing how the duration and maintenance of CR, as well as regulation of orexin and stress, may interact so that interventions can be designed towards optimal results for plasticity and mental health.

### 2.4. Impact of CR on Aging

*The Aging Brain and Brain Plasticity*. The aging brain is characterized by functional and metabolic changes, including an increased vulnerability to insults and disease [41]. These changes underlie the neuronal dysfunction associated with the cardinal feature of aging and cognitive decline, epitomized by impaired performance on tasks dependent on the hippocampus and associated networks [65, 66].

It is increasingly appreciated that severe neuronal loss does not drive age-related cognitive impairment but rather aging is characterized by numerous functional and structural alterations that together impair brain plasticity [65], such as a decrease in synaptic plasticity as evidenced by reduction in the ability of aged rats to sustain LTP [67]. Both levels of BDNF and its receptor TrkB decrease with age and these reductions correlate with impaired memory and dendritic spine density among hippocampal neurons [68]. In addition, AHN markedly declines with age and aging has also been reported to be a major contributor to the reduced proliferation of NSCs [66].

#### 2.4.1. Animal Studies

Table 3 outlines recent animal studies that have evaluated the effect of CR on brain plasticity in the context of aging.

Maintaining rodents on CR regimens prevents age-related declines in learning and preserves spatial and working memory [69, 70]. In addition, it is not only lifelong CR that exerts positive effects in combating age-related processes but also starting CR in midlife [71] or short exposures even at a late age [72–74] also entails beneficial effects. One of the mechanisms possibly underlying these effects is through maintaining the levels of NMDA receptors in the hippocampus, normally subject to reductions with age [44]. Preservation of these receptors enables reversal of age-related declines in LTP [75]. Many studies have reported that CR attenuates age-related declines in the levels of synaptic proteins [44, 76] particularly in the CA3 subregion of the hippocampus [44].

Mladenovic Djordjevic and colleagues demonstrate that lifelong CR enhances synaptic remodelling as evidenced by reversal of age-related declines in the expression of the presynaptic proteins synaptophysin, GAP-43, and *α*-synuclein in the hippocampus and cortex of rats [76]. CR also increases adult neurogenesis in young adult rats [77] and reverses age-related decline in neurogenic activity and significantly enhances survival of newborn glial cells in the DG in older mice [78, 79].

Another mediator of neural plasticity is neuropeptide Y, known not only for its function on the regulation of ingestive behavior [80], but also for its interaction with the cholinergic system for improved learning and memory [81], as well as for promoting hippocampal neurogenesis [82]. Recently, it has been reported that long-term 40% CR had multiple effects on the densities of neuropeptide Y receptor subtype densities throughout the brain [83]. For example, CR induced decreases in Y1-receptor density in the DG and reversed age-related declines in neuropeptide Y2-receptor density in CA2 subfield. These data lead the authors to suggest that long-term CR may exert specific effects of its own, in addition to combating age-related changes. In turn, changes in neuropeptide-Y-mediated signalling may impact brain circuits that regulate cognition and emotion [83].

#### 2.4.2. Human Studies

Whilst not conducted in an aging cohort, the National Institute of Aging CALARIE (Comprehensive Assessment of Long-Term Effects of Reducing Intake of Energy) study examined the effect of 6-month CR and CR in combination with physical exercise on cognitive function (*n* = 48; BMI: 25–30; age range: 25–50 yrs) [84]. A comprehensive array of cognitive tests of verbal memory, visual memory, and attention were conducted at baseline and at 3 and 6 months following intervention. During the trial, no improvements, but equally no deficits, emerged across these cognitive measures. These disappointing findings must be considered in context of a small sample size and limited statistical power.

The ENCORE (Exercise and Nutrition Interventions for Cardiovascular Health) study involved 124 participants (mean age: 52 ± 9 years) with elevated blood pressure, sedentary lifestyles, and a body mass index (BMI) greater than 25 [85]. Individuals adhering to antihypersensitive treatment combined with CR and exercise demonstrated significant improvements in both executive-function memory learning and psychomotor speed when evaluated at 4 months following intervention. The potentially synergistically interaction between CR, weight loss, and aerobic fitness likely underlies the neurocognitive improvements observed.

Witte and colleagues [234] report that adherence to a daily 30% CR regimen in a cohort of 50 healthy elderly subjects (mean age: 60.5 years; mean BMI: 28) improved performance on memory tests versus both a group with increased intake of PUFAs and the control AL group. In particular, verbal memory scores improved with a mean increase of 20% after 3 months of intervention in the CR group. Notably, the levels of BDNF did not change in the CR and PUFA groups. This is the first study to demonstrate the beneficial effects of CR on memory performance in an aged cohort. The negative BDNF finding does not definitively reflect an absence of BDNF involvement in mediating the effects of CR, as its levels may be altered in closer proximity to the start of the intervention or perhaps be revealed when related to other parameters such as neuroimaging [87]. In addition, whilst increased serum levels of BDNF have been linked to increased hippocampal volume and improved spatial memory in humans, even when controlling for variation due to aging [88], suggesting that circulating levels of BDNF may reflect levels of BDNF produced by the brain, BDNF is also produced by peripheral cells, tissues, and organs [89, 90]. As such, it remains an open question as to how accurately blood levels of BDNF reflect brain BDNF and its posited effects upon plasticity. Future studies should address longitudinal alterations in the levels of BDNF and other neurotrophins during the course of the CR intervention.

### 2.5. Impact of CR on AD

*Alzheimer's Disease (AD) and Brain Plasticity*. AD is an age-related and debilitating neurodegenerative condition epitomized by impoverished learning, memory, and executive function [91]. The pathognomonic features of AD include (1) the buildup of extracellular *β* protein (derived from cleavage of the amyloid precursor protein (APP) into diffuse plaques and (2) intranuclear aggregation of hyperphosphorylated forms of the microtubule and structural tau protein into neurofibrillary tangles [92].

Synapses are particularly vulnerable to impairment and irreversible damage upon exposure to *β*-amyloid and the synaptic formation is regarded as a key site in the initiation of the neurodegenerative process [93]. Human studies have reported compromised levels of BDNF and associated signaling in the preclinical stages of the disease and at postmortem [94, 95]. Alterations in the levels of hippocampal neurogenesis are increasingly regarded as an integral aspect of AD [96]. Notably, the putative role of nutrition in both contributing to and mitigating cognitive impairment in AD has emerged as a topic of increasing scientific and public interest [97–99].

#### 2.5.1. Animal Studies

Wu and coworkers report that 30% CR for 4 months in the transgenic mouse model of AD had profound effects on the pathophysiology of the disease, attenuating ventricle enlargement, hippocampal atrophy, and caspase-3 activation, as well as decreasing reactive gliosis and tau phosphorylation [100]. Moreover, upregulation of genes associated with neurogenesis and synaptic plasticity was found in the CR hippocampus, along with downregulation of genes associated with the expression of inflammatory markers. The combined modulation of disease pathology and enhancement of hippocampal neurogenesis by CR likely underlie amelioration of memory deficits as assessed by the novel object recognition and contextual fear paradigms in this study.

#### 2.5.2. Human Studies

The beneficial effects of CR on transgenic models of AD are consistent with epidemiological studies that report that high caloric diets are associated with an increased risk of developing AD [79]. For example, Luchsinger et al. observed that among those individuals in the Washington Heights-Inwood Columbia Aging Project cohort, a higher intake of calories and fats, the risk of developing AD was markedly elevated but only in individuals carrying the ApoE4 allele, a major genetic risk factor for AD [101]. In support of this, epidemiological studies reveal that those who habitually consume fewer calories have a reduced risk of developing AD (Gustafson et al.) [102]. In a recent population-based case-control study, Geda and coworkers report that a high calorific intake is associated with near twofold increase in risk of having mild-cognitive impairment [103].

## 3. Intermittent Fasting: Effects of Expanding Time between Meals

### 3.1. Background and Physiological/Molecular Mechanisms of IF

Intermediate or intermittent fasting (IF) refers to alternate periods of AL intake with complete or partial restriction of calories. Similar to CR, IF does not mean severe nutrient deprivation/starvation and all IF regimens are on a background of adequate vitamin and mineral intake [104]. IF has recently seen a surge in popularity through the advent of the “5,2” diet, which involves two days of complete or partial CR during a weekly period [105]. Importantly, IF does not necessarily reduce overall caloric consumption or lower bodyweight, since subjects may compensate for reduced intake during the restriction period by overeating on the AL phase, although in many studies implementation of IF results in a 20–30% reduction in caloric consumption over time [33, 35].

IF regimens have been demonstrated to induce a multitude of positive impacts on age-related diseases in animal studies, including the attenuation or prevention of diabetes-like phenotypes and cardiovascular disease, as well as increasing maximal lifespan [33].

At the molecular level, IF is thought to engage adaptive cellular stress response pathways and appears to engage many of the pathways described above for CR [38, 68].

### 3.2. Impact of IF on Brain Plasticity

The growing popularity of IF regimens among human subjects is supported by evidence that IF induces beneficial changes in animal studies; notably, many studies suggest that IF results in enhancement of brain plasticity and at cellular and molecular level with concomitant improvements in behavior (Table 1).

Maintaining rats on an IF regimen increased the resistance of hippocampal neurons to chemically induced degeneration [106, 107] and in experimental models of stroke [50]. Moreover, the neuroprotective effect of IF is also associated with preservation in learning and memory performance [50, 79]. Furthermore, the effects of IF following excitotoxic challenge associate with lower levels of corticosterone, leading not only to decreased hippocampal cell death, but also to increased levels of hippocampal BDNF and pCREB and reversal of learning deficits [107].

Increased survival of newly born cells in the DG, resulting in enhanced neurogenesis and gliogenesis, has also been reported as an effect of IF feeding in mice [108], as well as increased LTP and expression of the NMDA receptor subunit NR2B, resulting in enhanced learning [109].

Interestingly, IF but not CR for 20 weeks increases the resilience of hippocampal neurons to excitotoxic stress, suggesting distinct neuroprotective effects of IF [110].

### 3.3. Impact of IF on Mood/Anxiety

To our knowledge, no intervention or epidemiological study of the effects of IF on mood/anxiety in the human population has taken place; for this reason, we will focus this section on the promising findings from animal research (Table 2).

Whilst the majority of studies on the effects of fasting applied chronic protocols, a few have also investigated the outcomes of acute interventions. In this respect, a recent study has shown that 9 h fasting in mice leads to significant antidepressant effects [111]. Interestingly, additive effects were observed when the antidepressant drug imipramine was administered in conjunction with the fasting period. As proposed by the authors, these findings could be the start of a translation of acute fasting in conjunction with imipramine as a low-cost strategy to potentiate antidepressant effects in clinical practice. Furthermore, acute fasting led to an increase of the p-CREB/CREB ratio (p-CREB: phosphorylated CREB), a biological effect that is consistent with what has been found by some CR studies [46].

### 3.4. Impact of IF on Aging

#### 3.4.1. Animal Studies

Recent animal studies on the effects of IF upon aging are described in Table 3. Singh and coworkers aimed to test whether short-term late-onset exposure to an IF regimen could improve age-related declines in cognitive and motor functions, in association with possible changes in the expression of plasticity markers [112]. Old IF rats exhibited enhanced memory. Furthermore, these authors also evaluated Ca^2+^ signaling, a key regulator of synaptic plasticity, following short-term IF. In particular, they assessed the expression of serine/threonine protein phosphatase calcineurin (CaN), known for mediating the effects of Ca^2+^ signaling on synaptic plasticity, cell survival, and ultimately cognition [113], as well as the Ca^2+^-dependent protein kinase (CaM kinase), which plays a key role in synapse formation and neurotransmitter release, thus influencing neuroplasticity, learning, and memory [114, 115]. Notably, the expression of CaN and synaptophysin, which are known to significantly decrease with age in the CA3 and DG subregions of the hippocampus, was partially restored by the short-term IF and suggests recovery of loss of synapse density and concomitant increases in neurotransmission [112]. Given that IF decreased CaN levels and increased CaM expression in the hippocampus of aged rats, the potential beneficial effect of IF regimen on learning and memory likely involves the expression of synaptic proteins regulating calcium homeostasis [112].

Similarly, Mladenovic Djordjevic et al. report that IF induced elevated synaptophysin expression in the DG and CA3 [76]. These findings are supported by the similar effects of CR in mitigating age-related declines in synaptophysin levels [44, 116], suggesting that reduced levels of hippocampus stress, enhanced synaptic plasticity, and increased neurogenesis underlie the preservation of synaptic functionality associated with CR and IF regimens. IF has also been to shown to reverse the age-related impairments in the neuronal plasticity marker neural cell adhesion molecule (NCAM) [73].

#### 3.4.2. Human Studies

The aforementioned CALARIE study as well as studies in the cardiology field has provided proof of principle that dietary interventions based on reducing energy intake are not only safely tolerated by humans, but also elicit beneficial effects on a myriad of general health markers including insulin sensitivity, markers of oxidative stress, hypertension, and inflammation (see Weiss and Fontana, 2011, for a detailed review [40]).

Historically, a study by Vallejo later reanalyzed by Stunkard demonstrated that IF increased measures of healthspan and lifespan, proving its feasibility in human subjects [117, 118]. In support of these studies, Heilbronn and coworkers demonstrated that a 22-day IF regimen in a study of 16 nonobese humans (age range: 23–53) improved scores in the respiratory quotient, fat oxidation, and insulin sensitivity [119]. Together, these results suggest that IF is a feasible intervention in humans and is associated with beneficial effects in the metabolic profile; however, it is unlikely that such a regimen could be satisfactorily tolerated over a prolonged period owing to likely increase in aversive subjective states on the “off” day. The authors make a practical suggestion that the addition of a small meal on the fasting day improve the tolerability and adherence to IF.

In a recent study of 32 aged Malaysian men (age: 59.7 ± 6.3; BMI: 27), adherence to an approximate 25% CR diet in conjunction with two days/week of religious fasting resulted in marked reductions in scores on tests of tension, anger, confusion, and depression [120]. These positive changes upon affect occurred in conjunction with small reductions in weight, BMI, and percent body fat and support previous data from this group in a similar sized cohort where this same dietary intervention reduced scores on measures of depression [121].

It would be of great interest to further investigate the elicited effects of IF in a human study on measures of cognition, mood, and anxiety in conjunction with physiological markers of brain plasticity.

### 3.5. Impact of IF on AD

To our knowledge, no studies on the impact of IF upon AD date have been conducted with human subjects. As such, the findings discussed herein were generated from animal studies (Table 3).

Halagappa and colleagues utilized a triple transgenic mice model of AD, reporting that long-term maintenance of these mice (14 months) on either a 40% CR or IF diet, prior to the onset of the disease phenotype, rescued cognitive deficits [122]. The beneficial effects of CR on cognition were associated with significantly reduced levels of *β*-amyloid protein and phospho-tau. Intriguingly, IF appeared to protect neurons from injury despite the fact that there are no reductions in either A*β* or Tau, suggesting that this dietary approach may protect neurons downstream of A*β* and tau aggregation. Further to this, extensive *β*-amyloid deposition has been well documented in elderly persons in the absence of cognitive deficits [123]. Together, these data suggest that stimulation of adaptive stress responses is a possible mechanism by which cognition is preserved despite marked signs of disease pathology [43].

## 4. Dietary Content: Focus on Polyphenols as Potent Food-Derived Plasticity Inducers

### 4.1. Background and Physiological/Molecular Mechanisms of Polyphenols

Polyphenols are the most abundant antioxidants in the human diet and are present in a wide variety of fruits, teas, and consumed plants, including cocoa [124]. Besides their antioxidant action, polyphenols have been shown to enhance neuronal function as well as stimulate cerebral blood flow and neurogenesis [125]. Curcuminoids and flavonoids represent two of the main subtypes of polyphenols demonstrated to act upon brain function at the cellular and molecular level [124]. Curcumin, the most ubiquitous curcuminoid and active ingredient in the spice turmeric, has been consumed for medicinal purposes for thousands of years [126]. A distinctive feature of curcumin is the ability to modulate a multitude of signaling molecules and its varied properties, including being antibacterial, anti-inflammatory, chemotherapeutic, and neuroprotective [126].

Resveratrol is another naturally occurring polyphenol found in the skin of red grapes, nuts, and several other plants which has been extensively researched owing to its neuroprotective effects on the brain [86]. Resveratrol is a mimetic of CR, extending the maximal lifespan in yeast, worms, flies, and fish. In particular, both CR and resveratrol trigger overexpression of SIRT1, a NAD-dependent class III histone deacetylase that participate in transcriptional silencing of genes including those implicated in cell cycle regulation and lifespan extension [86]. The precise mechanisms underlying the neuroprotective role of resveratrol remain to be fully elucidated but it is likely that these beneficial effects result from a synergy of their antioxidant properties and SIRT1 activating capability [86].

Due to their well-documented effects on neural plasticity as well as upon cognition, mood/anxiety, and aging, this section will focus on findings from studies with curcumin and resveratrol.

### 4.2. Impact of Polyphenols on Brain Plasticity

In addition to their antioxidant properties, polyphenols have been reported to exert neuroprotective effects by directly modulating cellular pathways related to neuronal processes and synaptic plasticity (Table 1). In rodents, these include those that converge on the aforementioned CREB, a pivotal transcription factor linked to the expression of BDNF [127, 128], and other hippocampal plasticity related markers such as the expression of BDNF itself [128], glutamatergic receptors subunits (NMDA-NR2B), and polysialated neural cell adhesion molecule (PSA-NCAM: a marker of developing and migrating neurons) [129].

The classic activation of SIRT1 by resveratrol has been consistently demonstrated and to it novel players have been added which impact brain plasticity, such as the reduced expression of microRNAs miR-134 and miR-124 leading to upregulation of CREB levels and subsequently promotion of BDNF synthesis in the hippocampus [130]. However, SIRT-1 activation has also been suggested to negatively regulate neuronal differentiation in the adult hippocampus [131], suggesting other targets through which resveratrol might also trigger proplasticity effects, at least in the context of AHN.

An interesting report has provided evidence for novel insights on the mechanistic loop through which resveratrol might exert its effects on neural plasticity and, thereby, on cognitive improvement. After hypothesizing that orally administered resveratrol acts on the hippocampus through stimulation of the gastrointestinal tract (GI) leading to increased expression of insulin-like growth factor-I (IGF-I), Harada et al. showed that the enhancing effects of resveratrol on hippocampal angiogenesis, neurogenesis, and cognition were absent in calcitonin gene-related peptide (CGRP) knockout mice [132]. Sensory neurons of the mouse GI have been shown to transmit information to the hippocampus through the spinoparabrachial circuit, increasing the hippocampal levels of CGRP, which in turn induce local astrocytic production of IGF-I [133]. Given the abolishment of resveratrol-induced effects in CGRP^−/−^ mice, the authors propose that sensory neuron stimulation by resveratrol in the GI tract would lead to an increase of IGF-I production in the hippocampus, with consequent enhancement of hippocampal angiogenesis, neurogenesis, and, finally, cognitive function [132].

Possible synergistic effects of different dietary interventions on brain plasticity represent a promising field to translational neuropsychiatry. Interestingly, Dal-Pan and colleagues showed that long-term 70% CR or resveratrol supplementation for the same period (18 months) exerts similar beneficial effects for cognition in adult primates [134]. Whether the effects of CR and IF could be enhanced or in some contexts replaced by supplementation with polyphenols is thus an attractive field of research that could add to the potential of dietary interventions.

### 4.3. Impact of Polyphenols on Mood/Anxiety

To our knowledge, no functional studies on the impact of resveratrol or curcumin in the context of mood/anxiety have been conducted with human subjects; we therefore focus this section on findings from animal studies (Table 2).

Growing, although not unanimous [135], evidence suggests that the regular consumption of dietary polyphenols can lead to positive effects on mental health-related behaviors, a process probably involving brain plasticity [57].

The antidepressant effects of resveratrol have been consistently proposed by a number of recent studies. Chronic administration of resveratrol was able to prevent a wide range of detrimental effects on cognition induced by the classic unpredictable chronic mild stress model of depression [136], including reduction in serum corticosterone levels, increased levels of BDNF, phosphorylated extracellular signal-regulated kinase (pERK) and pCREB in the prefrontal cortex and hippocampus, and prevention of cognitive deficits [137]. Similar biological measures were found in nonstressed mice administered with resveratrol, which also presented with reduced depressive phenotype comparable with those of fluoxetine-treated animals [138]. Resveratrol has also been proposed to buffer prenatal stress in the adult offspring, likely through protective mechanisms against oxidative stress [139] and, of special relevance for neural plasticity, through enhancement of hippocampal BDNF and postnatal neurogenesis levels [140].

Interestingly, a number of studies point for a role of the serotoninergic system in modulating hippocampal plasticity in context of depression [141, 142]. Mice exposed to UCMS but receiving polyphenols extracted from tea leaves (tea polyphenols, TPs) daily from the 3rd week of stressors onwards presented with normalized depression-related phenotype [143]. Besides reversing the UCMS-induced expression of oxidative stress markers, TP administration was also capable of reversing the altered concentrations of serotonin and noradrenaline in the prefrontal cortex and hippocampus. Similarly, normalized levels of serotonin in the hippocampus and, furthermore, inhibition of the serotonin degrading enzyme monoamine oxidase-A (MAO-A) activity after UCMS exposure have also been shown to be biological effects of treatment with trans-resveratrol [144].

### 4.4. Impact of Polyphenols on Aging

#### 4.4.1. Animal Studies

Many different polyphenols have been reported to retard age-related declines in CNS function, cognition, and behaviour; recent reports in animal studies are outlined in Table 3. For example, blueberries, which are rich in anthocyanin and flavanols subset of polyphenols, are effective in persevering spatial working memory in aged animals [125], a process likely mediated by the marked increases in NSC proliferation in the DG of aged rats supplemented with these berries [145]. More recently, Rendeiro and coworkers report that dietary supplementation with flavonoids improves spatial working memory in association with increased expression of hippocampal BDNF [129].

Conboy et al. report that short-term (8 days) curcumin supplementation to aged rats enhanced PSA-NCAM expression in the DG and markedly improved both spatial learning and memory [146]. Dong et al. assessed the effects of 6- and 12-week curcumin-supplemented diet on hippocampal cellular proliferation, cognitive function, and transcriptional responses in aged rats [147]. Only in 12-week treatment did spatial memory improve, suggesting that prolonged curcumin consumption is required to prevent or slow down the decline of cognitive function with aging [147], contrary to the benefits of short-term exposure reported by Conboy and coworkers [146]. In addition, only the 12-week intervention enhanced AHN, suggesting that, at the dosage of curcumin used, the generation of new neuronal cells may require an accumulated effect of the active metabolites over a prolonged period [147].

Dong and colleagues provide further support for the relationship between prolonged exposure and its effects on brain plasticity, as evidenced by an exon array of hippocampal and cortical rat tissue that revealed differentially expressed genes with functions related to brain development, cognition, and neurogenesis but dependent on the length of curcumin treatment [147]. For example, expression of the NeuroD1 gene, integral for AHN and survival of neuronal progenitors [148], was increased following 6-week curcumin treatment. Additional genes altered by 6- and 12-week curcumin treatment included Wnt2, Nnat, Tiam1, and Unc5d which have diverse roles in neuron development. Further to this, 12-week curcumin treatment markedly upregulated genes implicated in synaptic transmission and memory formation, including Adcyl, Kit, and LPL in the hippocampus and Shank3, Cip98, Snip, and Nlgn2 in the cortex. Interestingly, it has been suggested that a reduction in the expression level of adenylyl cyclase I (Adcy1) in the hippocampus might greatly contribute to age-related defects in spatial memory [149]. Together, these results suggest that the beneficial effects of curcumin in improving cognition among aged rats are possibly explained by the enhancement of AHN and synaptic plasticity as facilitated by upregulation of development-associated genes in the brain [147].

#### 4.4.2. Human Studies

A number of epidemiological studies have reported that increased consumption of a number of different polyphenols improves various aspects of cognitive function in the aging population [150].

Among a population-based cohort of nondemented elderly Asian subjects (*n* = 1,010; mean age: 68.9 ± 6.8 years), multivariate analyses revealed that improved cognitive function was associated with those who regularly consume curry, and thus a large amount of the polyphenol curcumin, when compared to those who never or rarely consumed curry [151]. Similar results were reported in context of increased dietary flavonoid intake [152].

The recent report of 13-year long clinical study builds upon these findings by investigating the relationship between specific classes of polyphenols and cognitive performance in a cohort of 2574 middle-aged adults (age range: 35–60 at baseline) [153]. Utilizing participant dietary records regarding specific polyphenol consumption and a battery of cognitive tests, multivariate models revealed that increased intake of catechin, theaflavins, flavonols, and hydroxybenzoic acids was particularly associated with improved episodic memory and in some cases with preserved verbal memory. It must be noted that unexpected negative associations were also observed; for example, increased intake of the subclasses catechins; proanthocyanidins and flavanols were linked to poorer performance in tests of executive function. Whilst no mechanistic studies account for this negative association, it has been suggested that, under certain conditions, some catechins may exert multiple effects on the brain including a prooxidant action [154]. Nevertheless, together these encouraging studies build upon the molecular and cellular mechanistics reports conducted in animals to highlight the strong potential of dietary polyphenol intake to support preserved cognitive performance in the elderly population.

### 4.5. Impact of Polyphenols on AD

#### 4.5.1. Animal Studies

Polyphenols derived from multiple dietary sources are able to counteract cognitive deterioration and reduce neuropathology in different animal models of AD [155]. Recent animal studies also support a role for these compounds in promoting brain plasticity in context of AD (Table 3). For example, Dong and coworkers additionally report that 12-week curcumin treatment of aged rats resulted in markedly differential expression of genes thought to participate in both the AD neurodegenerative process and synaptic plasticity [147], such as the Cav1 gene implicated in alterations of cholesterol distribution in the AD brain [156], spatial memory formation [157], and age-related working memory decline [158].

Some interesting results were also generated by studies using a grape-derived polyphenolic preparation (GP), a mixture of proanthocyanidins (PACs), comprising catechin (C), epicatechin (EC), catechin gallate, and epicatechin gallate [159]. The potential therapeutic benefits gleamed from natural compounds containing GP and PAC are hindered by limited knowledge with regard to their metabolism and whether sufficient levels of these metabolites enter to brain to exert their effects [159]. Wang and coworkers aimed to bridge this knowledge gap by utilizing biosynthetic brain-targeted PAC metabolites and studying their pharmacokinetics in relation to their therapeutic effects upon cognitive deterioration in a transgenic mouse model of AD [159]. Animals were treated for 5 months starting at 7 months of age with different formulations of GP prior to the development of AD neuropathology and cognitive deficits. All three formulations were demonstrated to interfere with the initial protein-protein interaction of A*β*_1–40_ and A*β*_1–42,_ but only monomeric GP reduced the content of toxic oligomeric A*β* species and improved spatial memory retention. Of special note, the authors reported that the monomeric metabolite 3′-*O*-Me-EC-Gluc significantly increased the phosphorylation of CREB at differential sites. This study reveals for the first time that a brain-targeted metabolite derived from a polyphenol is capable of restoring synaptic plasticity in the AD-afflicted hippocampal formation.

Similarly, a recent study by Ho and colleagues evaluated the accumulation of polyphenol metabolites in the mouse brain of an AD-transgenic model following oral dosage with a Cabernet Sauvignon red wine (abundant in resveratrol) [160]. Using a dose that is equivalent to moderate daily wine consumption in humans, they report that one of the Cabernet Sauvignon brain-targeted metabolites, quercetin-3-0-glucuronide, reduced neuronal generation of *β*-amyloid peptides and prevented the formation of the toxic oligomeric species of this peptide [160]. Moreover, treatment with this metabolite markedly reversed AD-type deficits in hippocampal basal synaptic transmission and LTP, via a mechanism possibly dependent on activation of signaling pathways that result in phosphorylation of CREB.

Recent data from Hoppe et al. adds to the discussion of the neuroplasticity molecules possibly underlying the behavioral effects of polyphenols in context of AD [161]. Rats treated intraperitoneally with free or nanoencapsulated curcumin for 10 days did not show *β*-amyloid-induced cognitive impairment or typical decreases in both hippocampal synaptophysin and BDNF levels. Of special note, for future application in clinical practice, the nanoencapsulated curcumin administration was able to produce effects in a 20-fold lower dose, showing the potential of this technology for drug delivery to CNS targets.

The studies described above reveal for the first time that brain-targeted metabolite derived from polyphenols is capable of entering the brain, positively modifying AD neuropathology and is able to restore synaptic plasticity in the hippocampal formation and as such offers great potential as a novel therapeutic agent.

#### 4.5.2. Human Studies

A number of epidemiological studies have reported that increased consumption of a number of different polyphenols reduces the incidence and delays progression of AD [150]. For example, just as increased intake of the polyphenol curcumin improves cognitive performance in the Indian elderly population [151], it has been suggested that the low incidence of AD in this country is perhaps, in part, also attributable to the increased consumption of curcumin among its elderly population [127]. Similarly, a cross-sectional study of 1003 Japanese individuals over the age of 70 revealed that the consumption of more than or equal to 2 cups of green tea per day was associated with over a 50% reduction in cognitive deficit [162]. Several epidemiological studies have also indicated that moderate consumption of red wine is associated with a lower incidence of AD [163, 164].

There is a paucity of prospective clinical studies and trials investigating the therapeutic potential of polyphenols in AD [155]. However, results from the GuidAge study, a prospective prevention study on the impact of the* Ginkgo biloba* extract Egb761 on the conversion of elderly persons (*n* = 2854, age: 70 years and older at baseline) with memory complaints to AD, were recently reported [165, 166]. In this randomized control trial participants were required to twice daily consume a 120 mg dose of Egb761 for 5 years [166]. Disappointingly, long-term use of the polyphenol extract Egb761 did not reduce the risk of progression to AD when compared to placebo [166]. The authors noted that the number of dementia-related events in their study was much lower than expected resulting in reduced statistical power to detect any effect. Indeed, overrecruitment of healthy volunteers is a problematic issue in prevention trials, particularly in dementia research where baseline control individuals appear healthier and better educated than the general elderly population [167].

Despite the disappointing results from the clinical trial of the* Ginkgo biloba* extract, which can plausibly be explained by confounding factors, epidemiological studies clearly support the promising preclinical work implicating increased consumption of polyphenols in improved cognitive performance and lower incidence of AD.

## 5. Dietary Content: Focus on n-3 Fatty Acids as Potent Food-Derived Plasticity Inducers

### 5.1. Background and Physiological/Molecular Mechanisms of PUFA

Positive effects on brain health and function have also been shown as an outcome of PUFA-enriched diets as well as for those with high levels of fish and nut oils. Long chain essential PUFA, notably the omega-3 (n-3) fatty acids docosahexaenoic acid (DHA) and eicosapentaenoic acid (EPA), are fundamental to CNS function, considering the lipid-rich nature of the brain [168].

Omega-3 fatty acids are classified as essential, meaning that their levels depend on dietary intake, although their final functional availability also depends on other factors regulating metabolic events, such as polymorphisms of genes coding enzymes that convert short-chain to long-chain PUFAs [169]. Although fish is the major source of the EPA and DHA consumed (71%), these fatty acids are also contained in other foods, such as meat (20%), eggs (6%), and plant foods (such as leek and cereal-based products; 3%) [170].

The n-3 fatty acid DHA is an important structural component of neural cell membranes and is, thus, essential to appropriate neuronal functioning [171]. In particular, n-3 fatty acids can modulate cholesterol-induced reductions in membrane fluidity by displacing cholesterol from the plasma membrane [124]. This displacement leads to increased membrane fluidity, increased number of receptors, enhanced receptor binding and affinity, better ion channel functionality, and modulation of gene expression of proteins involved in signal transduction processes [172–175]. Together, these effects lead to improved neurotransmission and signaling and, therefore, to optimal cognitive functioning.

### 5.2. Impact of PUFAs on Brain Plasticity

Fatty acids have been implicated in enhancing brain plasticity and cognitive function in healthy, adult rodents (Table 1).

It has been shown that, in hippocampal slices of rats treated with EPA, LTP is significantly enhanced in CA1 neurons when compared with EPA-deficient hippocampal slices [176]. In addition, this same study showed that EPA applied* in vitro* was capable of decreasing cell death.

PUFA supplementation has also been associated with enhancement of AHN [178, 179], which could be one of the mechanisms underlying associated improvements in cognition and mood [180]. For instance, dietary supplementation with n-3 PUFAs restored neurogenic markers in the DG of a mouse model of systemic lupus erythematosus and Sjögren's syndrome, characterised by lower levels of AHN among other biological deficits [181]. Perinatal supplementation with n-3 PUFA also mitigated the impairment in working and short-term memory and altered fear response observed in rats as a consequence of sevoflurane-induced neurotoxicity, likely through decreased apoptosis and enhancement of hippocampal cell proliferation [182]. Perinatal supplementation with the DHA precursor *α*-linolenic acid (ALA) revealed a consistent improvement of hippocampal neurogenesis in the offspring, but only when the dam had been exposed to the enriched diet also during pregnancy [177], highlighting the importance of the appropriate timings of dietary interventions for maximized results.

### 5.3. Impact of PUFA on Mood/Anxiety

#### 5.3.1. Animal Studies

Recent studies focusing on the effects of PUFA on mood/anxiety in animals are presented in Table 2.

Rats exposed to an n-3-deficient diet during the gestational period and lactation presented with decreased brain levels of DHA and plasticity markers, such as BDNF and reduced activation of CREB in adulthood [184]. At the behavioral level, these animals exhibited increased anxiety-related phenotypes. Interestingly, similar biological changes but with additional decreases in expression of other plasticity markers such as GAP-43, Ca^2+^/calmodulin-dependent protein kinase II (p-CAMKii), and phospho-synapsin have been observed in rats initially maintained on n-3 fatty acid-rich diets during the gestational period until postnatal week 12, when a transition to a high-fat diet (low in n-3 fatty acids) was introduced [187].

Using a similar approach of switching rats from a PUFA-enriched or deficient diet to a western-like diet (WD) with higher percentage of fat and carbohydrates during the period of brain maturation, Tyagi et al. report increased anxiety-like behavior and decreased expression of the anxiety-related NPY-1 receptor, along with disruption of BDNF signaling in the frontal cortex of rats after mild traumatic brain injury (mTBI) [188]. mTBI is an established important risk factor for the development of psychiatric conditions such as posttraumatic stress disorder (PTSD); these results highlight the potential of healthy nutritional habits to counteract the lowered threshold for the onset of neuropsychiatric illness following mTBI. Indeed, n-3 fatty acid supplementation in rodents as a means to improve AHN is under analysis for the prevention of accidental injury-related PTSD [189].

A recent study further reinforces the idea that n-3 deficiency in rats might contribute to vulnerability to stress [186]. Conversely, increased levels of hippocampal BDNF associated with serotoninergic neurotransmission and antidepressant effects have also been observed in rats exposed to higher intake of fish oils [185].

#### 5.3.2. Human Studies

The potential antidepressant action of PUFA has been suggested to be true not only in animal studies, but also in human subjects [190–192].

Frequent fish consumption (and, in some cases, seafood intake as a whole) has been associated with decreased risk of depression and suicidal ideation [193], higher self-reported mental health status [194], and decreased prevalence of postpartum depression [195]. Moreover, the low concentrations of n-3 fatty acids in the blood of depressed patients [196, 197] also support the potential role of altered levels of PUFA in the development of depression. Furthermore, in patients diagnosed with unipolar depression, EPA treatment decreased symptoms of depression to a similar level to that of fluoxetine [198]. With special reference to translational potential, the effects of combined EPA and fluoxetine treatment were superior when compared to either intervention alone.

Besides their beneficial roles in unipolar depression, n-3 fatty acids have also been proposed to ameliorate depressive symptoms in context of bipolar disorder [199]. In addition, n-3 PUFA supplementation also decreased depressive and anxiety symptoms in early postmyocardial infarction patients [200], although some studies failed to show any improvement in depressive symptoms among this population [201].

Some authors argue that the antidepressant effects of PUFA are only identifiable within populations with severe, diagnosed depression [191, 201]. Whilst there is evidence to support this position in depression, it appears that this is not the case for anxiety. Indeed, n-3 fatty acid supplementation reduced symptoms in healthy, nonclinical anxious individuals [202].

Together, these findings identify PUFA supplementation as a potential translational tool in context of mood and anxiety disorders; however, depression and anxiety may arise in different contexts, comorbid with other medical conditions—psychiatric or not—and, thus, the exact doses, onset, and duration of PUFA supplementation for maximized results within different patient subsets requires further investigation.

### 5.4. Impact of PUFA on Aging

#### 5.4.1. Animal Studies

Supplementation of docosapentaenoic acid DPA, an intermediate molecule in the metabolism of DHA from EPA, preserves hippocampal function in the aged brain as evidenced by elevated measures of enhanced LTP when compared to control-fed counterparts [203]. Similarly, EPA has also been reported to preserve LTP in aged rats [67].

These findings complement recent data demonstrating a positive effect of dietary supplementation with PUFA on spatial memory in aged mice [203, 204]. Further to this, age-related decline in spatial memory performance is reflected by a decrease in c-Fos expression with age [205]. c-Fos expression reflects a neuronal response to extracellular signals such as growth factors and is triggered during action potentials [205]. Notably, provision of n-3 PUFA attenuates age-related declines in c-Fos expression [204].

Other studies, however, have reported little or no protective effects of DHA/EPA-enriched diets on aged-associated cognitive decline [174]. Such discrepancies in the literature are likely attributable to differences in length and/or composition of PUFA supplementation.

The positive effects of PUFA supplementation upon age-related cognitive decline in animals are likely governed by antioxidant [203] and anti-inflammatory mechanisms [204]. It will be of great interest to further explore how mechanisms related to brain plasticity also putatively contribute to PUFA-mediated reversal of age-related cognitive decline.

#### 5.4.2. Human Studies

Evidence from observational studies largely suggests that n-3 enriched diets may protect individuals from or slow down cognitive decline in the elderly population [206, 207]. For example, van Gelder and colleagues report results from a 5-year population-based prospective study of cognitive decline in relation to fish, EPA, and DHA consumption in a cohort of 210 nondemented elderly men (age range: 70–89) from the Netherlands [208]. Multivariate linear regression analyses revealed that, among individuals consuming fish, cognitive decline was significantly lower when compared to their counterparts who consumed no fish [208]. Further to this, the authors reported a dose-response relation between increased intake of EPA+DPA and less cognitive decline. Similarly, a 5-week crossover trail evaluating the impact of fish oil n-3 PUFA intake on cognitive performance revealed that PUFA supplementation in a Swedish cohort of 40 healthy middle-aged to elderly subjects (mean age: 63 ± 5 years) significantly improved working memory [209].

In a recent study, Titova and coworkers explored whether EPA and DPA intake impacted not only cognitive performance at 75 years, but also brain volume in a healthy cohort of 252 Swedish participants with a baseline age of 70 and a low dietary intake of these fatty acids at the start of study [210]. Multivariate analyses revealed that self-reported increases in EPA and DHA intake at 70 years positively associated with both cognitive performance and global grey matter volume [210].

It must be noted that not all clinical studies have reported a positive relationship between n-3 PUFA consumption and cognitive performance among the elderly population. For example, in a sample of 1025 elderly men (mean age: 68 years) from the Boston Veterans Affairs Normative Aging study, general linear models at both the cross-sectional and longitudinal levels did not reveal any significant association between fatty fish or n-3 PUFA intake and performance on a battery of cognitive tests after a 6-year follow-up [211]. In addition, a Cochrane review by Sydenham and colleagues reports that the pooling of data from three randomised-control trials of high-methodological quality also failed to show any benefit of n-3 PUFA supplementation on cognitive function in a sample of healthy elderly persons (age > 60 years), where n-3 PUFA supplementation occurred for at least 6 months (total *n* = 3536) [206]. It is possible that a protective effect of n-3 PUFA intake on cognitive decline may only be apparent with advancing age and marked cognitive decline.

On the whole, these findings do not support the data from the animal studies above, whereby n-3 PUFA markedly improves cognitive function in the aging brain. It is suggested by Sydenham and colleagues that trials of longer duration are required to better evaluate protective effects of PUFA supplementation on cognitive function in the elderly population [206].

### 5.5. Impact of PUFAs on AD

#### 5.5.1. Animal Studies

The most recent animal findings on the impact of PUFA on brain plasticity in AD models are described in Table 3.

Supplementing transgenic mouse models of the disease with a diet enriched mainly in DHA significantly lowered the synthesis of *β*-amyloid peptides and the formation of amyloid plaques [214]. With regard to brain plasticity, EPA supplementation has been shown to prevent the inhibition of LTP induced by *β*-amyloid [67].

Apolipoprotein *ε*4 (apoE*ε*4) is a major genetic risk factor for vascular dementia and sporadic AD [215]. Interestingly, when compared to the apoE*ε*2 and apoE*ε*3 isoforms, apoE*ε*4 functions inefficiently as a cholesterol transporter [216, 217], potentially impacting synaptogenesis and synaptic maintenance through altered release of cholesterol [218]. Further to this, maintaining mice with compromised apoE*ε*4 function on a fish-oil diet resulted in improved behavioral and cognitive performance [219].

It is posited that that enhanced beneficial effects can come from multidietary approaches, rather than from the supplementation of a single nutrient to patients at risk or diagnosed with AD [220]. In order to explore the mechanism by which multidietary nutrients putatively protect against AD, Jansen and colleagues investigated the effects on, among others, neural plasticity markers and associated behavior in apoE4-carrier and apoE knockout mice of a diet containing DHA, EPA, phospholipids, uridine monophosphate (UMP), choline, B vitamins, and antioxidants [213].

At the behavioral level, the multinutrient diet exerted anxiolytic effects for both apoE ko and wild-type mice; an important effect given that anxiety and restlessness are significantly correlated with impairments in activities of daily living in AD [221]. Further to this, Jansen and colleagues also reported that the multinutrient diet improved learning and spatial memory, but only in the apoE ko mice. This diet-associated improvement in cognition was independent of any change in synaptophysin-labelled presynaptic boutons and in the number of neuroblasts in the DG. These observations are in line with previous reports that failed to demonstrate an effect of n-3 PUFA diets on synaptophysin levels; instead, it appears that n-3 PUFAs exert their beneficial effects by improving synaptic function rather than synaptogenesis [219, 222]. With regard to the negative neuroblast finding, many studies reporting a positive effect of n-3 PUFA, folic acid, and vitamins and antioxidants on neurogenesis do so when making comparisons against nutrient-deficient animals [178, 223]. This suggests that the beneficial effects of dietary interventions might become apparent only when neurogenesis is severely compromised, as is the case during dietary deficiency but not in the apoE4 and apoE ko mice.

In a separate but similar study, Jansen and coworkers further affirm the anxiolytic effect of the PUFA-diet enriched with phospholipids, choline, folic acid, vitamins B6, B12, C, E, and selenium in a transgenic mouse model of AD [175]. Notably, n-3 PUFA on its own did not exert these effects, suggesting that it is the combination of additional nutrients which underlies the reductions in anxiety-like behaviour in this mouse model of AD. Both the PUFA only and PUFA plus additional nutrients diet had no effect in attenuating spatial learning, but mice in the latter group did show significant increases in doublecortin positive cells, suggesting that the multinutrient diet restored neurogenesis in this model of AD [175].

#### 5.5.2. Human Studies

Epidemiological studies suggest a role of n-3 PUFAs in slowing cognitive decline among elderly individuals and thus in reducing the incidence of AD [224]. For example, Morris and colleagues conducted a prospective population-based study of 815 initially healthy individuals (age range: 65–94) from the Chicago Health and Aging Project, reporting that after an average of 3.9 years follow-up the increased intake of DHA but not EPA was associated with a reduced risk of AD [225]. More recently, a study compared the plasma levels of EPA and DHA among individuals (age range: 55–91 years) with cognitive impairment but no dementia (*n* = 55), patients with AD (*n* = 19), and healthy volunteers (*n* = 61). Across the whole sample, dietary intake of n-3 fatty acids, plasma DHA, and plasma EPA positively predicted performance of delayed and verbal recognition memory [226]. Further research is needed to determine whether reduced levels of PUFA are secondary to AD or contributors to disease pathophysiology and cognitive decline. Interestingly, supplementation with n-3 PUFA has also been shown to exert beneficial effects on depressive symptoms and agitation in patients with mild to moderate AD [227], complementing many of the findings described in the animal studies section (Tables 2 and 3).

Intervention studies based on supplementation with n-3 PUFA, however, have proved to be disappointing, with many studies failing to show any protective effect against AD or cognitive decline except in some AD patients with very mild cognitive impairment [228]. The failure of these interventions, at least in part, are attributable to the testing in patients already with mild to moderate dementia, a too late time point in the pathophysiological course of AD [229]. In addition, these negative results may reflect intrinsic limitations in the design of these trials such as the preponderance to recruit healthier elderly participants who are more motivated to adhere to instructions to eat healthier and undertake exercise more, thus confounding results by not being a truly representative sample of the target population [167].

As described above, many other dietary components/regimens possess diverse properties to promote brain plasticity. Considering the complementary roles of polyphenols and PUFA, it is not surprising that the specific multinutrient diets containing a combination of these factors have been developed to mitigate risk factors associated with AD [230]. Indeed, two randomized, double-blind controlled clinical trials reported that daily consumption of such multinutrient component diets improved memory performance in patients with mild AD [230, 231].

In summary, whether the lack of agreement between mechanistic studies in animals and observational data in the trial setting represents poor efficacy of n-3 PUFA in mitigating AD-induced cognitive decline or intrinsic limitations of trial design remains an open question [228].

## 6. Discussion

### 6.1. Animal and Human Studies: Bridging the Gap

**Figure 1:**
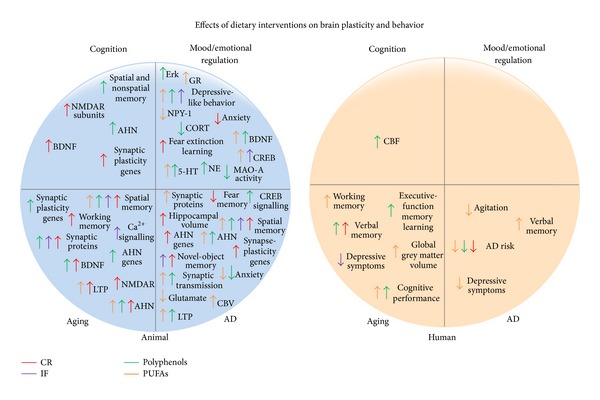
Different dietary interventions in animal and human studies are believed to modulate various aspects of brain plasticity and in turn influence behaviour. Animal studies provide the vast majority of our current mechanistic understanding of the potential mechanisms by which dietary interventions impact brain plasticity. Further mechanistic studies aiming to fill the gap in our understanding of how diet can modulate plasticity and promote mental health in human populations are clearly needed. Moreover, additional intervention studies are also required to demonstrate efficacy, enabling the safe translation of such dietary interventions into clinical practice or incorporated into our daily lifestyles to enhance brain health/function and well-being. In red, effects induced by CR; in purple, effects induced by IF; in green, effects induced by supplementation with polyphenols; in orange, effects induced by PUFAs. AD: Alzheimer's disease; AHN: adult hippocampal neurogenesis; BDNF: brain-derived neurotrophic factor; CBF: cerebral blood flow; CBV: cerebral blood volume; CORT: corticosterone; CR: calorie restriction; CREB: cAMP responsive-element binding; 5-HT: 5-hydroxytryptamine; IF: intermittent fasting; GR: glucocorticoid receptor; LTP: long-term potentiation; MAO-A: monoamine oxidase A; NMDAR: N-methyl-D-aspartate receptor; NE: noradrenaline; NPY-1: neuropeptide Y type 1 receptor; PUFAs: polyunsaturated fatty acids.

The marked contribution of poor dietary habits to the ever growing prevalence of neurological and psychiatric diseases is increasingly recognized [232]. Many of the animal studies and indeed some of the human studies described above demonstrate that diet-induced brain plasticity offers tremendous potential in promoting emotional and cognitive well-being across a variety of contexts. In particular, diet influences multiple aspects of brain plasticity, including neurodevelopment, neurotrophins, neurogenesis, synaptogenesis, and ultimate activity at the brain network level (Figure 1). Together these processes underlie and influence cognitive and mood/emotional processing, thus positioning diet as a key modulator of brain structure and function.

Importantly, diet-induced brain plasticity is putatively a low-cost and effective means to protect against the debilitating effects of psychiatric and neurological disorders. This viewpoint is strongly supported by many of the cellular and molecular animal studies discussed which together indicate that in addition to being a noninvasive intervention diet entails a “broad spectrum of action” [233]. Notably, the wide range of cellular and molecular mechanisms engaged by dietary factors and regimens such as CR, IF, polyphenols, and PUFAs can be advantageous for the treatment of neurological disorders characterized by diffuse pathology and deficits across multiple cellular domains. This indicates that diet has strong translational potential and is further supported by the promising results from epidemiological studies that have shown that these dietary factors entail improvements in emotional and cognitive domains in humans [153, 209, 234].

We now discuss some of the other factors involved in the so-called translational gap, namely, important caveats and obstacles that must be considered if diet-induced brain plasticity is to be successfully implemented in the clinical setting.

### 6.2. Combining Diet and Exercise

Feeding and exercising are complementary aspects of regulating energy balance that have influenced the evolution of the modern brain over thousands of years [124]. Given that the brain poses the largest demand on oxygen consumption, it is not surprising that energy metabolism has a profound influence on brain function [127]. In particular, both food consumption and physical activity stimulate mitochondrial activity and thus energy provision to the brain which in turn modulates the signaling pathways linked to neuronal function and brain plasticity [127].

In addition to its role in enhancing brain plasticity, BDNF has also been implicated in modulating brain energy metabolism, as evidenced by studies that demonstrate that perturbed BDNF signaling can manifest in metabolic disorders such as obesity [45]. Together, these studies reveal that BDNF plays a key role in both brain energy metabolism and plasticity, demonstrating a strong and influential relationship between diet, exercise, and brain function [45, 127].

Similar to diet, exercise entails a “broad spectrum of action” and also effectively promotes brain plasticity through the increases of neurogenesis, neurotrophins levels, and synaptic plasticity [235]. Indeed, it is possible that exercise potentiates the health-promoting effects of diet components and vice versa at the cellular and molecular levels. For example, it has recently been demonstrated that exercise works in tandem with a DHA-enriched diet to enhance cognitive function [236]. In particular, exercise appears to act on mechanisms that preserve DHA on the plasma membrane and in turn enhance neurotransmission [233]. In addition, the concurrent effects of DHA diet and exercise engage BDNF-mediated synaptic plasticity [237].

Further to this, in a rodent model of traumatic brain injury (TBI), the combination of DHA supplementation and voluntary exercise restored membrane homeostasis to counteract the detrimental effects of TBI on many parameters of synaptic plasticity and cognition [238]. With regard to synaptic plasticity, exercise greatly enhanced the action of DHA supplementation on levels of BDNF and TrkB activation following TBI [238]. Together, these data reveal a strong and novel interaction between diet and exercise, whereby aspects of these lifestyles intersect at the molecular level under pathological conditions to promote brain plasticity [238].

Similarly, exercise and flavonoid-enriched diets together promote the elevation of genes that promote brain plasticity whilst decreasing expression of markers known to compromise this plasticity, including those related to inflammation and cell death [239]. Moreover, exercise has also been shown to markedly reduce the effects of a diet rich in saturated fats through the counteracting of declines in BDNF-mediated synaptic plasticity in the hippocampus [240].

The combination of exercise and CR is particularly noteworthy. In this paradigm, typically, 12.5% of the energy restriction comes from adherence to a restricted diet and the other 12.5% comes from increased energy expenditure via exercise [33]. The principal advantage of combining CR with exercise is that individuals may find it easier to comply with energy restriction if this split between CR and exercise-induced expenditure [241]. Therefore CR plus exercise may well prove viable and effective means of promoting brain plasticity. Some studies, however, have reported that CR plus exercise does not elicit positive changes to health other than those elicited by CR. For example, animals maintained on an 80% CR plus exercise regimen demonstrated no significant changes in oxidative stress, in proinflammatory markers [242, 243], or upon extension of life span [244].

### 6.3. Diet-Induced Brain Plasticity in the Modern World

In a thought-provoking study, Agrawal and Gomez-Pinilla reason that “unhealthy dietary habits are difficult or almost impossible to completely eliminate” and that this grim reality necessitates the concurrent supplementation of healthier dietary components to popular diets as a strategy to counteract metabolic dysfunction in the brain and ultimately to protect mental health [245]. In particular, these authors investigated whether a DHA-enriched diet could counteract the debilitating effects of insulin resistance on brain plasticity. Deficiency of n-3 exacerbated a decline in spatial memory performance in proportion to the intensity of insulin resistance. Further to this, the authors observed that rats fed with the n-3 diet were able to maintain the ratio of n-6/n-3 within the normal range, even in the presence of fructose, indicating preservation of membrane fluidity and in turn promotion of synaptic plasticity and cognition [245].

Further to this, n-3 deficiency decreases phosphorylation of CREB and reduces the levels of two markers of synaptic plasticity—synapsin I and synaptophysin [245]. Importantly, fructose consumption potentiated this effect. Together, data from this study implies that adequate levels of DHA are particularly necessary under challenging conditions such as the metabolic syndrome, and given the abundant consumption of sugars in western society, proper consumption of DHA emerges may be an important means to preserve brain plasticity.

### 6.4. The Challenge of Imaging Brain Plasticity

Whilst cognitive behavioral testing in human subjects and animals is a necessary and appropriate means of assessing brain plasticity following dietary intervention, there is a pressing need to relate cognition, mood/emotionality, and behavior to* in vivo* structural and dynamic quantitative assessments which will enable direct inference of a diet-induced effect on the brain [125]. We have described above several examples whereby AHN appears to be enhanced following dietary intervention but investigation of both the dynamics and functions of AHN in humans has remained challenging owing to the absence of accepted macroscopic neuroimaging readouts [246].

Brain imaging studies utilizing fMRI and transcranial Doppler ultrasound have revealed that efficient cerebral blood flow (CBF) is integral to optimal brain function as evidenced by marked reductions in CBF among individuals with impoverished cognitive performance and patients diagnosed with dementia [247, 248]. As such, the observation that 1-2 hour infusion of subtypes of polyphenol markedly and rapidly increases CBF measures in human subjects is of great interest [249, 250]. Further to this, intervention with a flavonol-rich drink derived largely from cocoa increased blood flow, as measured by the blood oxygen level-dependent (BOLD) signal on fMRI, in certain regions of the brain as well as modifying the BOLD response during a task switching test [249]. Whilst these studies primarily focus on the flavanol subset of polyphenols (e.g., cocoa), it is highly plausible to suggest that other polyphenols, particularly those rich in other flavonols such as grapes and blackcurrants, may also impact positively CBF [125].

More definitive proof of diet-induced brain plasticity will require such fMRI measures to be compared with changes in regional grey matter volume as well as biomarkers of NSCs using proton NMR spectroscopy [125]. As such, by combining a multimodal neuroimaging, biochemical, and behavioral approach, the effects of diet on brain plasticity will be better illuminated in a mechanistic, dynamic, and quantitative way. Despite these challenges, the diet field can take inspiration from a recent study by Voss and colleagues that assessed the effects of exercise on brain plasticity in combination with fMRI investigation [87].

### 6.5. Animal versus Human Brain Plasticity

Perhaps more important than the technical challenges described in Section 6.4, with regard to trying sensitively capturing dynamic processes such as neurogenesis in the human brain, is to acknowledge possible fundamental neurobiological and functional differences related to brain plasticity when extrapolating from findings in animals to humans. Taking adult hippocampal neurogenesis as an example, whilst the levels of this process appear comparable in middle-aged rodents and humans [251], it is now appreciated that age-related declines in rodents are potentially more pronounced than in humans [251, 252]. Such an observation has implications for dietary regimens known to specifically increase neurogenesis in older animals, as there is a possibility that these same regimens will not elicit the same magnitude of increases because the baseline levels of neurogenesis are potentially higher in elderly humans.

These remarks, however, do further emphasize the need for improved* in vivo* temporal and spatial resolutions of NSCs in both animals and humans, in order to facilitate robust comparisons as well as quantitatively determine changes in the levels of NSCs during and following a dietary intervention.

### 6.6. Genetic Interaction

Diet-gene interactions are pivotal in mediating the effects of diet-induced brain plasticity, with subsequent effects on brain (patho)physiology [253]. Indeed, single nucleotide polymorphisms, copy number variants, modifications to the epigenome as well as the activity of microRNAs, and long noncoding RNAs can all modify the effects of nutrition on gene expression [253].

Intriguingly, a recent study reported that 9 dietary polyphenols appear to counteract the modulation of differentially expressed miRNAs in apoE knockout mice, suggesting that polyphenols engage similar mechanisms [254]. Whilst the precise functions of these miRNAs remain to be elucidated as well as the extent of the influence of the ApoE genotype on modulating the effects of polyphenols on miRNA expression, this type of study clearly demonstrates that diet-gene interaction is yet another important player and requires further investigation [253].

More generally, animal studies afford the possibility to investigate the impact of interventions on a genetically homologous cohort. In contrast, extrapolating findings from such animal studies to heterogeneous humans necessitates caution owing to varying genetic backgrounds of participating subjects. Moreover, this heterogeneity also limits the generalizability from one population to another owing to differences in factors related to lifestyle and ethnicity.

### 6.7. Supplementation and BBB Permeability

DHA has a multitude of actions at the cellular and molecular level, making it difficult to ascribe recommended levels of intake [124]. Indeed, a number of studies have demonstrated that a varied range of n-3 intake can have beneficial effects on mental health [255]. It must be emphasized that these effects are dependent on the length of supplementation and are likely to be influenced by baseline levels [124].

Current recommendations issued by the dietary supplement industry for resveratrol are 20 mg per day but it must be stressed that these recommendations are not specific to enhancing synaptic plasticity or exerting an effect on brain function [255]. As such, clinical applications of dietary polyphenols supplementation in context of emotional health or neurodegenerative disease demands further research [124].

Owing to the effects of gut enzymes on the metabolism of polyphenols, their metabolites exhibit a wide range of bioavailability [256]. The ability of the metabolites of polyphenols to cross the blood brain barrier (BBB) is a major determinant of their bioavailability to brain cells [127]. Importantly, many polyphenols have been claimed to exert neuroprotective effects but many polyphenols have also been demonstrated to be incapable of crossing the BBB, suggesting that their suggested benefits to the brain may arise via indirect or other pathways [127]. As reviewed above, the identification and profiling of bioavailability and pharmacokinetics of specific metabolites of polyphenols by Wang et al. 2012 [159] and Ho et al. 2013 [160] that not only penetrate the BBB but also act on AD neuropathology as well as promote neuronal plasticity provide two considered examples of attempts to translate the observed epidemiological benefits of grape-derived products and red wine in AD into clinical applications.

### 6.8. Ethical and Socioeconomic Considerations

It must be noted that the long-term effects of CR (over 1 year) in elderly persons remain to be elucidated and as such caution must be followed before engaging in a CR regimen [97]. Whilst there are encouraging signs from studies in animal models regarding the benefit of CR and/or IF to mitigate or delay the onset of AD, it must be noted that malnutrition is a common problem among the elderly; as such, dietary recommendations will have to take into consideration this background [98].

In addition, it is not known whether the putative benefits of CR are confined to the restriction period or long lasting after the intervention, of which the length also remains to be determined [97]. More likely, CR will be the most effective when undertaken in conjunction with exercise or intellectual and environmental enrichment [97]. Given these concerns, IF appears a more feasible option for humans rather than lifelong CR which may prove to be difficult to adapt to over a lifetime [112]. Further to this, reports have shown that long-term CR may entail detrimental effects on cognitive ability in middle- and old-aged rats, attributable to low blood glucose levels [257].

Socioeconomic position (SEP) as determined by parameters such as income, education, and occupation is an important determinant of dietary intake with higher SEP generally related to better diet quality [258]. Interestingly, SEP is also associated with differences in cognitive function across life [259, 260]. Like diet, animal evidence suggests that conditions simulating SEP modulate neurobiological mechanisms implicated in brain plasticity and the structural and functional changes in response to these life experiences [261].

Parrott and colleagues investigated whether SEP influences the impact of diet quality on cognitive function. They observed that cognitive benefits of adherence to a “prudent” (healthy) dietary pattern were garnered regardless of SEP. These benefits were also differential; for example, higher adherence to this diet was associated with less decline in those with low SEP, whereas it was associated with better performance among those with high SEP [262]. The authors propose that these findings are consistent with the concept of cognitive reserve, whereby individual differences in lifestyle may allow for more successful accommodation of age-related brain changes by protecting the amount of neural substrate or the efficiency of brain networks mediating performance [263].

## 7. Conclusion

Whilst much remains to be elucidated at the mechanistic level in both animal and humans, the current literature provides robust evidence for the essential roles played by dietary factors in promoting brain plasticity. Animal studies reveal that a multitude of dietary regimens and components increase the levels of adult hippocampal neurogenesis and neurotrophins as well as acting to enhance synaptic function. These cellular and molecular changes likely underlie marked improvements in cognitive performance and emotional regulation across a number of disease contexts in animals maintained or supplemented on these different dietary regimens. Moreover, this inference is supported by the largely positive epidemiological studies, positioning diet as an important factor in disease risk, progression, and severity.

Taken together, we argue that whilst the field must be mindful of the “gap” between the effects of diet across animal and human studies it is by no means insurmountable and perhaps smaller than first envisaged. Importantly, future dietary intervention studies must take into account timing of treatment onset and its duration; synergistic/additive effects with multiple dietary components and exercise; longitudinal and multimodal measurements; the longevity of diet-induced effects. It can be concluded that an appropriate dietary intake, encompassing the aforementioned considerations—food amount, frequency, and content—should be actively encouraged as a cost-effective, noninvasive, and broad spectrum public health initiative for both the prevention and amelioration of neuropsychiatric disorders.

## Figures and Tables

**Table 1:** Effects of diet on brain plasticity and cognition in animal studies from 2010 onwards.

Model	Dietary factor	Intervention	Cellular and molecular mechanisms	Effects on behavior	Conclusion/proposed mechanism	Reference
5-week-old male Wistar rats	EPA-E	1.0 mg/g/day for 8 weeks via gavage	↑LTP in CA1, ↑hippocampal p85*α*	N/A	EPA exerts neuroprotective effects via synaptic plasticity enhancement	[176]

4-month-old obese and nonobese male Wistar rats	60% CR	10 weeks	↑hippocampal NR2A and NR2B levels in CR obese rats; ↓MDA levels in all CR groups	N/A	CR prevents oxidative stress, protecting NMDAR subunits 2A and 2B in obese rats which can result in ↑LTP and synaptic plasticity	[49]

3-month-old female California mice	80% CR	8 weeks in SD or LD photoperiod	No CR-induced changes in hippocampal synapsin I or GFAP	↓performance in LD mice in reversal learning (Barnes maze)	Effects of CR on spatial learning are photoperiod dependent	[48]

6-month-old BCKO mice	70% CR	5 weeks	↓LTP in CR controls but not in BCKO	↑memory and ↓aggressiveness in CR controls but not in BCKO	CR effects depend on CREB-1 by its regulation of sirtuin transcription in neuronal cells	[46]

12–14-week-old male SHRSP rats	70% CR	28 days or 28 days of CR + EX training	↑hippocampal BDNF in CR + EX rats	↑cognition (MWM) in CR + EX rats	CR + EX act synergistically to upregulate BDNF and prevent cognitive decline in SHRSP rats	[51]

~3-month-old male Sprague-Dawley rats	IF or ADX + IF or IF + KA or ADX + IF + KA	14 weeks	↓CA2/CA3 cell loss by IF and ADX + IF after KA insult; ↑BDNF and pCREB in ADX + IF only	Attenuated KA-induced learning deficit in a T-maze task by ADX + IF	IF protects hippocampal neurons against KA insult; IF effects are ↑under lower levels of CORT	[107]

10–12-week-old male C57BL/6 mice; CGRP−/− mice	RES	20 mg/L orally administered once daily for 3 weeks	↑hippocampal CGRP, IGF-I and IGF-I mRNA; ↑angiogenesis and AHN; no effects in CGRP−/− mice	↑spatial learning (MWM); no effects on CGRP−/− mice	RES stimulates sensory neurons in the GI tract, ↑IGF-I production and promoting angiogenesis and AHN, thereby ↑cognition	[132]

38-month-old male grey mouse lemurs	70% CR or RES	70% CR or RES (200 mg/kg/day) for 18 months	N/A directly; similar levels of serum CORT	↑working memory (CSA); ↑spatial performance (CPT) only in RES group	CR and RES seem to induce similar benefits on cognitive functions in an adult primate by probably activating striatoprefrontal circuits and hippocampus	[134]

10-week-old male Wistar rats	Blueberry	2% for 7 weeks	Activation of ERK1/2; ↑CREB; ↑pro- and mBDNF in the hippocampus; ↑BDNF mRNA in the DG and CA1	↑spatial learning in a delayed nonmatch task (8-arm maze)	Flavonoid-rich blueberries ↑spatial learning in young healthy rats, likely through activation of ERK-CREB-BDNF pathway in the hippocampus	[128]

8-week-old male Wistar rats	Flavonoids	8.7 mg/day or 17.4 mg/day for 3 weeks	↑PSA-NCAM in the DG and NMDA-NR2B in the hippocampus; ↑ERK/CREB/BDNF signaling, and ↑activation of the Akt/mTOR/Arc pathway	↑spatial memory acquisition and consolidation	Flavonoid-induced improvements in learning and memory might involve upregulation of PSA-NCAM and NMDA-NR2B	[129]

10-week-old C57BL/6J female mice and PND19 male offspring	ALA or ALA-def	Gestation and/or lactation	ALA during gestation + lactation ↑cell proliferation and neuronal differentiation in the DG of PND19; ALA-def ↑apoptosis	N/A	ALA is required in both fetal and postnatal stages for enhanced AHN in offspring	[177]

Effects of different proneural plasticity dietary interventions (CR, IF, and polyphenolic/fatty acid supplementation) on brain function and cognition in recent animal studies (2010 onwards). ADX: adrenalectomy; ALA: *α*-linolenic acid; BCKO: brain CREB knockout mice; BDNF: brain-derived neurotrophic factor; CGRP: calcitonin gene-related peptide; CORT: corticosterone; CR: calorie restriction; CREB: cAMP responsive-element binding; CSA: continuous spontaneous alternation task; DG: dentate gyrus; EPA-E: ethyl eicosapentaenoic acid; ERK 1/2: extracellular signal-related kinase 1/2; EX: exercise training; GI: gastrointestinal tract; IF: intermittent fasting; IGF-I: insulin-like growth factor-I; KA: kainic acid; LD: long day; LTP: long-term potentiation; mBDNF: mature form of BDNF; MDA: malondialdehyde; MWM: Morris water maze; N/A: not assessed; NMDAR: N-methyl-D-aspartate receptor; p-CREB: phosphorylated CREB; proBDNF: precursor form of BDNF; PSA-NCAM: polysialylated neural cell adhesion molecule; RES: resveratrol; SD: short day; SHRSP: stroke-prone spontaneously hypertensive rats.

**Table 2:** Effects of diet on brain plasticity in animal studies of mood/anxiety from 2010 onwards.

Model	Dietary factor	Intervention	Cellular and molecular mechanisms	Effects on behavior	Conclusion/proposed mechanism	Reference
ICR strain male mice	Acute fasting	3 h, 9 h, and 18 h or 9 h + i.p. injection of IMI (30 mg/kg) or 9 h + i.p. injection of IMI (30 mg/kg) + DOI (5 mg/kg)	↑ratio of p-CREB/CREB in 9 h fasting mice	↓depressive-like behavior (FST) in 9 h fasting mice, which was more pronounced in 9 h + IMI. Effects reversed by DOI	Antidepressant-like effects of acute fasting possibly occur via ↑p-CREB/CREB ratio, and additive effects with IMI via modulation of 5-HT_2_ receptors	[111]

**C57BL/6J mice 7-8 ** **weeks of age**	CR	Moderate 10–15% CR for 3 weeks after CR, a subset of mice was refed either with a high-fat or chow diet AL	CR ↑stress-induced corticosterone levels, ↓BNST CRF levels and ↑BNST CRF promoter methylation ↑MCH and orexin among post-CR mice transitioned to high-fat diet	CR ↑depressive-like behaviour (TST) ↑binge eating of palatable high-fat foods after CR MCH receptor-1 antagonist ↓total caloric intake in post-CR mice on high-fat diet	Moderate CR reprogrammes pathways involved in regulating stress responsivity and orexigenic drives. Management of stress during diet may be beneficial in long-term maintenance	[64]

20–22 g male ICR mice	Trans-RES	10 mg/kg, 20 mg/kg, 40 mg/kg or 80 mg/kg via gavage, acute	↑hippocampal 5-HT and ↓MAO-A activity (40 or 80 mg/kg)	↓depressive-like behavior (FST: 20, 40, and 80 mg/kg; TST: 40 and 80 mg/kg)	Antidepressant-like effects of trans-RES might be related, among others, to modulation of the 5-HT system	[142]

180 g–200 g male Wistar rats	RES or UCMS + RES	80 mg/kg, i.p., once daily for 5 weeks	Prevented UCMS-induced ↑serum CORT, and ↓BDNF, pERK, and pCREB levels in the PFC and hippocampus	Prevented UCMS-induced cognitive deficits (MWM; NORT)	RES prevents UCMS-induced cognitive impairment partly via normalizing serum CORT levels and upregulating BDNF, pERK, and pCREB in the PFC and hippocampus	[137]

200–250 g male Wistar rats	CUR or UCMS + CUR	10 mg/kg via oral gavage, once daily for 3 weeks	N/A	Prevented UCMS-induced depressive phenotype (SP; OFT)	CUR exerts antidepressant effects partially by preventing UCMS-induced ↑of TNF-*α*, IL-6, and NF-*κ*B in the PFC and hippocampus	[183]

18–22 g male Kun-Ming mice	TPs or UCMS + TPs	25 mg/kg or 50 mg/kg by gavage once daily for 3 weeks from 3rd week on of UCMS	Reversed hippocampal and prefrontal cortex alterations of 5-HT and NE	Reversed UCMS-induced depressive-like behavior (FST, TST, SP, and OFT)	Antidepressant action of TPs might be related to modulation of monoaminergic responses and ↑antioxidant defenses	[143]

22–25 g male Kun-Ming mice	RES or FLU	20 mg/kg or 40 mg/kg or 80 mg/kg (RES); 10 mg/kg (FLU), i.p., once daily for 21 days	↑BDNF and ERK levels in the hippocampus and PFC, ↓serum CORT	↓depressive-like behavior (FST and TST)	Antidepressant-like actions of RES are probably mediated by modulation of the HPA axis, BDNF, and Erk levels in the hippocampal and PFC	[138]

190 g–200 g male Sprague-Dawley rats	Trans-RES	10 mg/kg, 20 mg/kg, 40 mg/kg or 80 mg/kg via gavage 30 min before the chronic stress for 21 days	↑5-HT levels in the frontal cortex, hippocampus, and hypothalamus (80 mg/kg); inhibited MAO-A activity in the frontal cortex and hippocampus (10–80 mg/kg)	↓depressive-like behavior (SP and shuttle box test: 40 and 80 mg/kg)	Antidepressant-like effects of trans-resveratrol involves, among others, the regulation of 5-HT levels and MAO-A activity	[144]

8-9-month-old C57BL/6J and SIRT1 mutant mice	RES	Intraventricular injection of RES (5 *µ*g/*µ*L for a week)	↑LTP in CA1; ↑BDNF and CREB in hippocampal slices; ↓miR-134 and miR-124; effects blocked in SIRT1 mutant mice	↑fear memory (contextual and tone-dependent memory test); effects blocked in SIRT1 mutant mice	RES exerts its effects via regulation of microRNA-CREB-BDNF mechanism, likely in a SIRT1 dependent way	[130]

3-4-month-old female Wistar rats and PND40 offspring	RES or RES + CRS	10 mg/kg orally administered throughout pregnancy	↑hippocampal DCX and BDNF	N/A	Resveratrol neuroprotects against prenatal stress likely via AHN improvement	[140]

280–300 g female pregnant Sprague-Dawley rats; 15-week-old male offspring	n-3 diet or n-3 def	Gestation, lactation, and postnatal week 15	n-3 def ↓levels of DHA, NPY-1, BDNF and CREB; ↑GR in the frontal cortex, hypothalamus and hippocampus	n-3 def ↑anxiety-like behavior in the OFT and EPM	DHA deficiency during gestational and postnatal development ↓brain plasticity and compromises brain function in adulthood	[184]

10-week-old virgin female Wistar rats and PND90 male offspring	FO	Adaptation period (15 days), mating (8 days), pregnancy (21 days), and nursing (21 days)	↑hippocampal and cortical BDNF; ↑hippocampal 5-HT	↓depressive phenotype (FST); effects reversed by 5-HT1A antagonist	n-3 PUFA exert antidepressant effects likely via increase in hippocampal 5-HT transmission	[185]

6-month-old male Wistar rats	n-3 diet or n-3 def or n-3 diet + CRS or n-3 def + CRS	25 g/day from weaning to 3 months; 20 g/day until 6 months; CRS for 21 days	N/A	n-3 def ↓locomotor activity induced by CRS and ↑startle response	n-3 deficiency may contribute to vulnerability to stress	[186]

280–300 g female pregnant Sprague-Dawley rats; 12-week-old male offspring	DHA or HFD	DHA = from gestation to postnatal week 15; HFD = DHA from gestation to postnatal week 12 + HFD until 15 weeks	Switch from DHA to HFD ↓DHA levels, NPY, BDNF, pCREB, GAP-43, pCAMKii, and p-syn expression in frontal cortex, and hippocampus	Switch from DHA to HFD ↓locomotor activity in the OPF and ↑anxiety-like behavior in one of the measures of the EPM	Transition from DHA to HFD ↓plasticity markers and is associated with increased anxiety	[187]

8-week-old BAFF Tg	PUFAs	12 weeks	PUFAs restored AHN and LTP	N/A	PUFA can restore AHN in autoimmune mouse model	[181]

Female Sprague-Dawley rats and PND7 offspring	n-3 PUFAs (dam) or n-3 PUFAs (dam) + sevoflurane (offspring)	from pregnancy to PND14 (n-3 PUFAs); 6 h at PND7 (sevoflurane)	n-3 PUFAs attenuated sevoflurane-induced neuronal apoptosis; ↑cell proliferation in the DG	n-3 PUFAs restored fear response to footshock and ↑working and short-term memory (MWM)	PUFA can improve altered memory and fear response in sevoflurane-treated rats via ↓apoptosis and ↑AHN	[182]

280–300 g female pregnant Sprague-Dawley rats; 15-week-old male offspring	n-3 diet or n-3 def or n-3 diet + WD or n-3 def + WD or n-3 diet + WD + FPI or n-3 def + WD + FPI	n-3 diet or n-3 def during brain maturation; WD for 6 weeks at 8 weeks of age	n-3 def + WD disrupted BDNF signaling (TrkB, CaMKII, Akt, and CREB) and ↓NPY-1 in the frontal cortex; more pronounced after FPI	n-3 def + WD ↑anxiety-like behavior (EPM); more pronounced after FPI	n-3 def + transition to WD might lower the threshold for neurological disorders via BDNF and NPY-1 signaling disruption	[188]

Effects of different proneural plasticity dietary interventions (CR, IF, and polyphenolic/fatty acid supplementation) on brain function and behavior in in recent animal studies (2010 onwards) of mood/anxiety. AHN: adult hippocampal neurogenesis; BDNF: brain-derived neurotrophic factor; BNST: bed nucleus of the stria terminalis; CORT: corticosterone; CREB: cAMP responsive-element binding; CRF: corticotropin-releasing factor; CRS: chronic restraint stress; CSA: continuous spontaneous alternation task; CUR: curcumin; DCX: doublecortin; DG: dentate gyrus; DHA: docosahexaenoic acid; DOI: serotoninergic 5-HT_2A/2C _receptor agonist (±)-1-(2,5-dimethoxy-4-iodophenyl)-2-aminopropane hydrochloride; EPM: elevated plus maze test; 5-HT: 5-hydroxytryptamine; 5-HT1A: 5-hydroxytryptamine type 1A receptor; FLU: fluoxetine; FO: fish oil-supplemented diet; FPI: fluid percussion injury; FST: forced swimming test; GAP-43: growth-associated protein 43; GR: glucocorticoid receptor; HFD: high fat diet; IL-6: interleukin 6; IMI: imipramine; i.p.: interaperitoneal injection; LTP: long term potentiation; MAO-A: monoamine oxidase-A; MCH: melanin-concentrating hormone (MCH); MWM: Morris water maze; N/A: not assessed; NE: noradrenaline; NF-*κ*B: nuclear factor kappa B; NORT: novel object recognition test; NPY-1: neuropeptide Y type 1 receptor; n-3 def: n-3 deficient diet; n-3 diet: n-3 adequate diet; OFT: open field test; p-CAMKii: Ca2+/calmodulin-dependent protein kinase II; pERK: phosphorylated extracellular signal-regulated kinase; PFC: prefrontal cortex; p-syn: phospho-synapsin; PUFA: polyunsaturated fatty acid-enriched diet; RES: resveratrol supplementation; SP: sucrose preference; TNF-*α*: tumor necrosis factor alpha; TPs: tea polyphenols; Trans-RES: trans-resveratrol; TrkB: tyrosine kinase receptor B; TST: tail suspension test; UCMS: unpredictable chronic mild stress; WD: western diet.

**Table 3:** Effects of diet on brain plasticity in animal studies of aging and AD from 2010 onwards.

Model	Dietary factor	Intervention	Cellular and molecular mechanisms	Behavioral effects	Conclusion/proposed mechanism	Reference
F1 male Fischer 344 × Brown Norway (F344 × BN) rats	CR	Lifelong 40% CR from 4 m of age in young (10 m) versus old (29 m) rats	No effect upon spine number, density, or morphology in CA3	N/A	CR alters synaptic protein levels rather than number to compensate for synaptic loss	[212]

Male Wistar rats	CR	Lifelong CR comprising 50% of the mean daily intake of the AL group every 2nd day for middle-aged (12 m), aged (18 m), and old (24 m) rats	Counteract age-related alterations of the presynaptic proteins SPH, GAP-43, and *α*-syn	N/A	CR ↑synaptic remodelling and ultimately changes synaptic function and/or structure in the absence of a change in synapse number	[76]

Rat	EPA or DPA	Groups of young (3-4 m) and aged (20–22 m) rats treated with EPA and DHA for 56 d	Deficits in LTP are reversed in the aged rats that received EPA or DPA	Deficits in spatial learning are reversed in the aged rats that received EPA or DPA	Preservation of cognitive function following n-3 PUFA supplementation in aged animals is supported by complementary anti-inflammatory, antioxidative, and metabolic effects	[203]

Male Sprague-Dawley rats	CUR	Short-term (6 w) and long-term (12 w) curcumin-supplemented diet to old rats (15 m)	12 w intervention ↑neurogenesis; 12 w treatment markedly upregulated genes implicated in synaptic transmission and memory formation, for example, Cav1 gene—implicated in both cholesterol metabolism in AD and synaptic plasticity	Only 12 w treatment ↑spatial memory	Beneficial effects, explained by the enhancing of adult neurogenesis and synaptic plasticity, may require an accumulated effect of the active metabolites over a prolonged period	[147]

Transgenic mouse model of AD (Tg2576)	Grape-derived polyphenolic preparation comprising a mixture of PAC	5 m treatment starting at 7 m of age (before AD neuropathology/cognitive deficits). 10 d treatment to assess pharmacokinetics and bioavailability. Tg2576 mice aged 22–24 m used to assess the effect of PAC metabolites on LTP	↑levels of metabolites from PAC monomers were detected in the plasma and brain of mice. Biosynthetic PAC monomer metabolite ↑LTP in the CA1 region and ↑phosphorylation of CREB at [Ser133]	Only the monomeric PAC improved spatial memory retention	Brain-targeted metabolite derived from a polyphenol is capable of restoring synaptic plasticity in the AD-afflicted hippocampal formation	[159]

Male Wistar rats	IF	Old rats (at 70% of their lifespan) maintained on short-term (3 m) IF regimen	Partial restoration of expression levels of SPH and calcineurin in the CA3 and DG	Attenuation of age-associated impairments in spatial learning and motor coordination	Beneficial effect of IF regimen on learning and memory is mediated by expression of synaptic proteins regulating calcium homeostasis	[112]

Embryonic 14–16 d cortico-hippocampal neuronal cultures derived from Tg2576 AD mice	Cabernet Sauvignon (red wine derived poylphenol)	Cells were treated with varying doses of the polyphenols equivalent to moderate daily wine consumption in humans	Caberent Sauvignon brain-targeted metabolite quercetin-3-0-glucuronide reverses AD-type deficits in hippocampal basal synaptic transmission and LTP, via activation of cellular modulators of CREB protein signalling pathways.	N/A	Quercetin-3-O-glucuronide in the brain may simultaneously modulate multiple independent AD disease-modifying mechanisms, including enhancing synaptic plasticity	[160]

ApoE4-carrier and ApoE knockout mice	Multinutriet diet Fortasyn (FC), containing DHA, EPA, phospholipids, uridine monophosphate (UMP), choline, B vitamins, and antioxidants	At 2 months of age, the mice were put on either control or FC diet for the remainder of the experiment. Behavioral testing was performed at 9 m. MR imaging was performed at 11 m	No change in the levels of synaptophysin and neurogenesis MRS revealed decreased levels of glutamate in both the apoE knockout and wild-type mice increase in CBV in a region of mid-brain in the apoE ko and wild-type mice fed	Anxiolytic effect on apoE ko and wild-type mice. Improved learning and spatial memory performance only in the apoE knockout mice	n-3 PUFAs seem to exert their beneficial effects by improving synaptic function rather than by increasing synaptogenesis. Increase in CBV possibly reflects improvement in brain perfusion	[213]

Male transgenic mouse models of AD (A*β*PP-PS1)	Diet enriched with DHA, EPA, and UMP (DEU diet) or diet enriched with DHA, EPA, UMP as well as phospholipids, choline, folic acid, vitamins B6, B12, C, E, and selenium (FC diet)	Feeding the diets started when the mice reached the age of 2 months and was maintained for the remainder of the experiment. Animals underwent behavioral testing at 11 months of age and subsequently MRS measurements at 12 months of age	Both diets had no effect on reversing declines in the levels of N-acetylaspartylglutamate (tNAA) FC but not the DEU diet had a significantly higher amount of doublecortin positive cells FC diet ↓hippocampal levels of unbound choline-containing compounds in wild-type and transgenic animals	FC diet exerts an anxiolytic response Both DEU and FC diets had no effect on attenuating spatial learning or memory deficits	The FC diet was more effective than the DEU diet in counteracting neurodegenerative aspects of AD and enhancing processes involved in neuronal maintenance and repair. Specific multinutrient diets can influence AD pathophysiology, including enhancing brain plasticity.	[175]

20-month-old male Sprague-Dawley rats	CR	40% CR for 12 months	Prevented age-induced decrease of NPY5 receptors in CA2	N/A	Regulation of NPY receptors in the old brain by long-term CR protects neural circuits involved in cognition, emotion, and feeding functions	[83]

300–350 g male Wistar rats	CUR or CUR-LNC or A*β* + CUR or A*β* + CUR-LNC	50 mg/kg (CUR) or 2.5 mg/kg (CUR-LNC) once daily for 10 days beginning 4 days after A*β*	Prevented A*β*-induced ↓of hippocampal SPH and BDNF	Prevented A*β*-induced cognitive impairment (NORT)	Neuroprotective effects of CUR on A*β*-induced memory impairment could be linked to Akt/GSK-3*β* pathway activation and ↑BDNF expression	[161]

18-month-old male Wistar rats	Pure anthocyanins, pure blueberry powder, or pure flavanols	2% for 6 weeks	↑BDNF levels and ↑BDNF mRNA expression in the hippocampus (anthocyanins)	↑spatial memory in an alternation task	Flavonoids likely exert causal effects on the cognitive improvement induced by flavonoid-rich foods	[129]

Effects of different proneural plasticity dietary interventions (CR, IF, and polyphenolic/fatty acid supplementation) on brain function and behavior in recent animal studies (2010 onwards) of aging and Alzheimer's disease (AD). *α*-Syn: *α*-synuclein; BDNF: brain-derived neurotrophic factor; CBV: cerebral blood volume; CR: calorie restriction; CREB: cAMP responsive-element binding; CUR: curcumin; CUR-LNC: curcumin in lipid nanocapsule; DEU: n-3 fatty-acid enriched diet; FC: n-3 fatty-acid enriched diet supplemented with additional factors such as polyphenols; GAP-43: growth-associated protein 43; IF: intermittent fasting; LTP: long-term potentiation; NORT: novel object recognition test; NPY: neuropeptide Y; NPY-5: neuropeptide Y type 5 receptor; PAC: proanthocyanidins; p-CREB: phosphorylated CREB; SPH: synaptophysin.
